# Validating a therapy-oriented complication grading system in lumbar spine surgery: a prospective population-based study

**DOI:** 10.1038/s41598-017-12038-7

**Published:** 2017-09-18

**Authors:** David Bellut, Jan-Karl Burkhardt, Dania Schultze, Howard J Ginsberg, Luca Regli, Johannes Sarnthein

**Affiliations:** 1 University Hospital Zurich, Department of Neurosurgery, Zurich, Switzerland; 2 University of Zurich, Zurich, Switzerland; 3 St. Michaels Hospital, Division of Neurosurgery, University of Toronto, Toronto, Canada; 4 ETH, Zurich Neuroscience Center, Zurich, Switzerland

## Abstract

The aim of the present study was to validate a therapy-oriented complication grading system in a well-defined neurosurgical patient population in which complications may entrain neurological deficits, which are severe but not treated. The prospective patient registry of the Department of Neurosurgery, University of Zurich provides extensive population-based data. In this study we focused on complications after lumbar spine surgeries and rated their severity by Clavien-Dindo grade (CDG). Analyzing 138 consecutive surgeries we noted 44 complications. As to treatment, CDG correlated with the length of hospital stay and treatment cost. As to patient outcome, CDG correlated with performance and outcome (McCormick). The present study demonstrates the correlation between outcome scales and the CDG. While the high correlation of CDG with costs seems self-evident, the present study shows this correlation for the first time. Furthermore, the study validates the CDG for a surgical subspecialty. As a further advantage, CDG registers any deviation from the normal postoperative course and allows comparison between surgical specialties.

## Introduction

Degenerative lumbar spine disease is extremely common and is increasing in prevalence with rising life expectancy^1,2^. Degeneration of the lumbar disc is one of the most frequently encountered pathologies in neurosurgical practice leading to conditions like disc herniation, spinal canal stenosis and degenerative spondylolisthesis. Other lumbar spine pathologies seen in neurosurgical practice include discitis/osteomyelitis, fractures, congenitial anomalies and spinal tumors. Symptoms related to these pathologies account for a large amount of morbidity and disability in modern society. Low back pain is the third most common symptom of any kind reported by patients and is also frequently found in the above-mentioned conditions^1,2^. Surgical treatment options for some of these pathologies include procedures such as micro-discectomy, laminectomy, hemi laminectomy or fenestration for spinal canal decompression as well as instrumented fusion with or without decompression and/or transforaminal or posterior lumbar interbody fusion (T-LIF/P-LIF). These procedures have been found to help lower the burden of disease and improve quality of life in selected patients. Most of the above-mentioned surgical procedures are considered routine. Nevertheless, with degenerative, tumorous and infectious conditions being more frequently diagnosed in elder people, these patients often suffer from other medical comorbidities, which make them prone to medical and surgical complications even when undergoing “minor” or “routine” procedures.

Documentation, rating and follow-up of complications is necessary to gauge treatment decisions and to improve informed consent and patient outcomes. Furthermore, with an aging population and worldwide struggle with rising health care costs, complications are an important socioeconomic factor. Understanding frequency, type and consequences of complications are the first steps to take in order to reduce them. Several complication scores have been introduced for use in Neurosurgery. The Clavien-Dindo complication grading system (CDG) was introduced in 2004 to rate surgical complications in general and is therapy-oriented, i.e. it rates the clinical measures required in response to complications^3^. The score has since been widely used in different areas of surgery such as general surgery, or orthopedic surgery^4,5^ and thus facilitates comparisons between surgical specialties.

The application of a therapy-oriented grading system to neurosurgery, however, is not obvious because severe complications may occur, e.g. hemiplegia, which do not entrain therapy and would simply be graded as “any deviation from the postoperative course”. We therefore set out to evaluate CDG and its correlation with outcome, length of stay and treatment costs in a well-defined neurosurgical patient population in a prospective, single-center study.

## Patients and Methods

### Patients

We included all consecutive patients who underwent surgical procedures for lumbar spine pathologies in our department between January 2014 and April 2015. Inclusion criteria included hospitalization in the Department of Neurosurgery, University Hospital Zurich, surgical treatment for lumbar spine pathology and age >18 years. Exclusion criteria were treatment for other neurosurgical pathologies and outpatient treatment only.

The prospective collection of patient data and outcomes through the registry of the Department of Neurosurgery was approved upfront by the local ethics review board (Kantonale Ethikkommission KEK-ZH 2012–0244) and it was registered internationally at clinicaltrials.gov (NCT01628406). Data reporting follows the STARD guidelines. Patients signed informed consent for surgery and data collection and all analysis and data collection was in accordance with relevant guidelines and regulations.

### Measures

The patients’ clinical symptoms, surgical characteristics, complications and outcome were evaluated from the surgeon’s perspective at admission, discharge and at 6 weeks follow-up. All data were entered prospectively into the department’s patient registry^6^, which had been installed in 2013 to monitor the quality of surgical treatment and patient care.

Treatment costs were taken from the clinical information system of our hospital (KISIM, www.cistec.ch) and normalized using the standard reimbursement rate for the uncomplicated surgical treatment of a lumbar disc herniation (treatment cost of 1).

General performance was rated using the modified Rankin scale (mRS, Table 1) and the Karnofsky performance status scale (KPS, Table 2). As a scale specific to spinal procedures we used the modified McCormick scale^7^ (1 neurologically intact; 2 mild motor or sensory deficit; functional independence; 3 moderate deficit, limitation of function; 4 severe motor or sensory deficit, dependent; 5 paraplegia or quadriplegia). Last follow-up was either at discharge or at 6 weeks postoperatively. We define as improvement the difference in scales between admission and last follow-up.

**Table 1:** Modified Ranking Scale (mRS).

Grade	Definition
0	No symptoms
1	No significant disability, despite symptoms; able to perform all usual duties and activities
2	Slight disability; unable to perform all previous activities but able to look after own affairs without assistance
3	Moderate disability; requires some help, but able to walk without assistance
4	Moderately severe disability; unable to walk without assistance und unable to attend to own bodily needs without assistance
5	Severe disability; bedridden, incontinent and requires constant nursing care and attention
6	Death

**Table 2:** Karnofsky Performance Status.

Grade	Definition
100	Normal; no complaints; no evidence of disease
90	Able to carry on normal activity; minor signs or symptoms of disease
80	Normal activity with effort; some signs or symptoms of disease
70	Cares for self; unable to carry on normal activity or to do active work
60	Requires occasional assistance, but is able to care for most of their personal needs
50	Requires considerable assistance and frequent medical care
40	Disabled; requires special care and assistance
30	Severely disabled; hospital admission is indicated although death not imminent
20	Very sick; hospital admission necessary; active supportive treatment necessary
10	Moribund; fatal processes progressing rapidly
0	Dead

Complications entered our analysis if they were noted at discharge or at 6 week follow-up. The severity of complications was rated by the Clavien-Dindo grading system^3^ (CDG, Table 3). For ease of handling, we report only the worst complication of each patient.

**Table 3:** Clavien Dindo Grading System (CDG) and number of cases.

Grade	Definition	Number of cases (%)
	No complication	93 (67%)
1	Any deviation from the normal postoperative course without the need for pharmacological treatment or surgical, endoscopic, and radiological interventions. Allowed therapeutic regimens are drugs as antiemetics, antipyretics, analgesics, diuretics, electrolytes, and physiotherapy. This grade also includes wound infections opened at the bedside.	19 (14%)
2	Requiring pharmacological treatment with drugs other than such allowed for grade I complications. Blood transfusions and total parenteral nutrition are also included.	16 (12%)
3	Requiring surgical, endoscopic or radiological intervention	
3b	- General anesthesia not required	4 (3%)
3b	- General anesthesia required	6 (4%)
4	Life-threatening complication requiring ICU management	0
4a	- Single organ dysfunction	
4b	- Multi organ dysfunction	
5	Death of a patient within 30 days of surgery	0

### Statistical analysis

To describe variation within the data, we present percentages together with the 95% confidence intervals (CI) based on the binomial distribution. We used non-parametric statistical methods for hypothesis testing. The non-parametric Spearman’s rank correlation was used to analyze correlation between CDG and KPS, duration of hospital stay and treatment cost. Statistical analyses were performed with custom scripts in Matlab (Version 2014b). Statistical significance was accepted at the p < 0.05 level.

## Results

### Patient group

We included 138 patients (82 male, 59%) with mean age 56 ± 17 years (range 17–89 years). Twenty-one patients (15%) had a history of previous spine surgery. The clinical characteristics are given in Table 4.

**Table 4:** Patient characteristics.

Number of patients	138
Male/Female	82 (59%)/56 (40%)
Age (years)	56 ± 17
Primary spinal pathology:	
- Disc herniation	78 (57%)
- Spinal canal stenosis	25 (18%)
- Spinal instability	16 (12%)
- Intradural tumor	9 (7%)
- Other	10 (7%)
Length of stay in hospital (days)	7 ± 5

### Clinical outcome

Surgery improved the mean clinical status in our patient population between admission and discharge. We found significant improvement of clinical symptoms measured with the McCormick scale between admission and discharge (p < 0.001) and between discharge and 6 weeks follow up (p < 0.001). Rankin scale equally improved by the time of discharge in comparison to admission (p < 0.001) and showed another significant improvement within the first 6 weeks after surgery (p < 0.05). Karnofsky performance scale improved in the above mentioned intervals with p < 0.001 and p < 0.001 respectively.

### Complications

**Figure 1:**
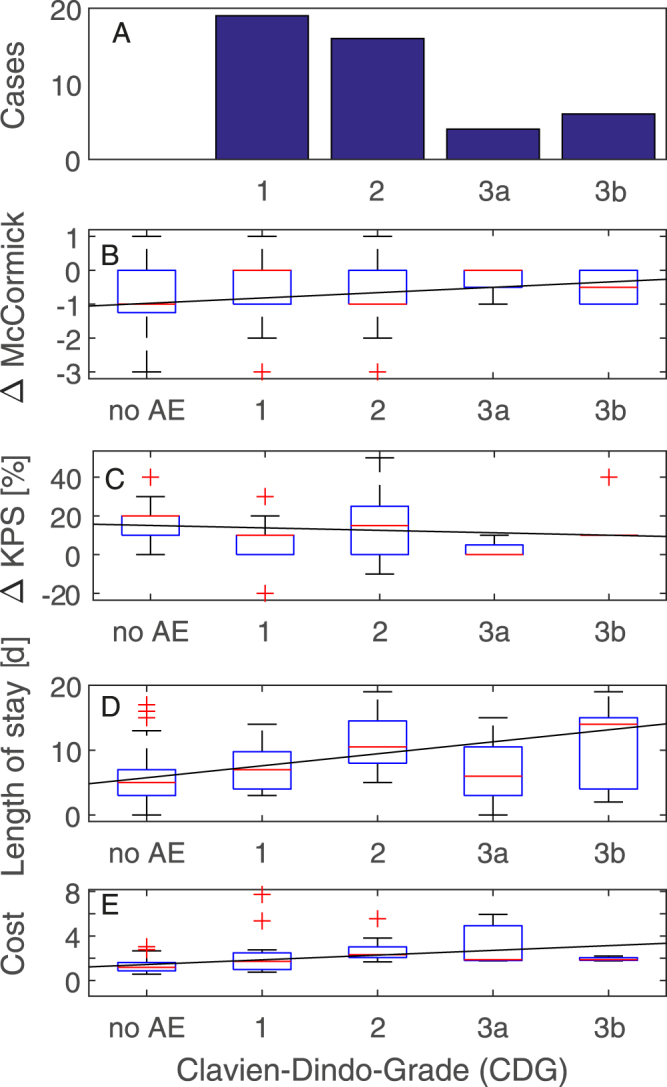
Effect of the severity of complication noted at discharge or at 6 week follow-up. (**A**) Distribution of the 45 complications registered up to the first 6 weeks after discharge. (**B**) McCormick Grade. Lines are linear least square fits (rho = 0.24, p = 0.005, 0.16 McCormick points per increment of CDG). (**C**) Karnofsky Performance Status Scale (KPS, rho = −0.19, p = 0.025, −1.3 KPS points per increment of CDG). (**D**) Length of stay in hospital (rho = 0.40, p < 0.001, 1.8 days per increment of CDG). (**E**) Relative costs of treatment and treatment cost (rho = 0.45, p < 0.001, 0.4 cost units per increment of CDG).

Any deviation of the normal postoperative course (CDG ≥ 1) was registered in 44/138 = 32% CI [24% 40%] cases (Table 1). The majority of complications (35/44 = 80% CI [65% 90%]) were treated without invasive treatment (CDG 1 and CDG 2, Fig. 1A).

Neurosurgical complications included temporary new neurological symptoms (3.6%) such as minor weakness, numbness or tingling, wound healing complications (3.6%), dural tear (1.4%) and one case of CNS infection (0.7%). The complete list of complications at discharge is given in Table 5.

**Table 5:** Type of the worst complication per patient at discharge and at 6 weeks follow-up.

Complication	Number of patients	surgical	medical	related	serious
New neurological symptoms or unexpected or new pain (temporary)	12 (11 unexpecteded postoperative pains, 1 new neurological symptoms)	12	0	12	12
Cardiovascular complications	6	0	6	0	4
Wound healing complication	5	5	0	5	5
Urinary tract infection	4	0	4	4	4
Recurrent herniated disc	4	4	0	4	4
Dural tear	4	4	0	4	0
Fever	2	0	2	0	2
Epileptic seizure	1	0	1	0	1
Anemia	1	1	0	1	0
Meningitis	1	1	0	1	1
Allergic reaction to dressing	1	1	0	1	0
Subileus	1	0	1	1	1
Exacerbated diabetes	1	0	1	0	0
Osteoporotic fracture	1	0	1	0	1
Foot swelling	1	0	1	1	0
Total	45	28	17	34	35
Percentage of N = 138	33%	20%	12%	25%	25%
95% confidence interval	25–41%	14–28%	7–19%	18–33%	18–33%

If a complication was disabling, life-threatening or prolonging hospitalization it was rated as serious.

Regarding the therapy, the presence of any complication in a patient was associated with longer hospitalization (median 9 *vs* *.* 5 days, p < 0.001) and higher treatment cost (median 1.6-fold p < 0.001). As expected from a therapy-oriented definition of complication severity, the CDG grade correlated significantly length of stay (rho = 0.40, p < 0.001, 1.8 days per increment of CDG, Fig. 1D) and treatment cost (rho = 0.45, p < 0.001, 0.4 cost units per increment of CDG, Fig. 1E).

Regarding the clinical outcome, the median KPS at last follow-up was higher without than with a complication (90 vs. 80, p = 0.0003, Mann-Whitney U-test). KPS at last follow-up also correlated with CDG with rho = −0.33, p < 0.001, −4.1 KPS points per in increment of CDG. However, patients with a lower KPS at admission may be more prone to complications. We therefore analyzed the improvement between admission and last follow-up. The improvement in KPS was correlated with CDG (rho = −0.19, p = 0.025, −1.3 KPS points per increment of CDG, Fig. 1C). Also the improvement in McCormick grade was correlated with CDG (rho = 0.24, p = 0.005, 0.16 McCormick points per increment of CDG, Fig. 1B).

## Discussion

Analyzing data from a prospective neurosurgical patient registry, we selected a well-defined group of consecutive patients with lumbar spine surgeries and found a significant correlation between the severity of a complication and treatment cost, performance, and neurological improvement. The correlation with cost or length of stay seems to be self-evident from a therapy-oriented definition of complication severity as in CDG, but regarding the treatment costs this has never been shown before. Furthermore, the present study validates the CDG for the first time in a well- defined patient population of a surgical subspeciality. Surgery for spinal pathologies are among the most frequent procedures in neurosurgery and orthopedics which makes structured analyzing, documenting and reducing incidents of complications in the field even more important.

In an earlier report of the registry^6^, we analyzed the whole patient population operated in our department with respect to feasibility and usefulness of the method. In the present study we focused on the validation of the CDG in a homogenous neurosurgical patient group different from the fields of medicine where CDG has previously been applied. Furthermore, we took into account the possibility that patients with a worse clinical state at admission may be more prone to complications and analyzed the improvement, i.e. the difference in scales between admission and last follow-up.

### Advantages of CDG

At first glance, the complication rate with CDG may appear higher than in studies using other grading scales. As an advantage, CDG comprehensively registers any deviation from the normal perioperative course and not only severe complications, which prevents underreporting. Conversely, other complication grading systems may underestimate the rate by neglecting complications unrelated to surgery or adverse events that were not treated. A comprehensive grading scale is clinically relevant as can be seen from the correlation with clinical outcome.

As a further advantage, CDG is a widely studied instrument especially in general surgery, which has been proved reliable in a wide number of studies and also some surgical subspecialties^3^ and thus allows comparing complications rates and types of surgical procedures beyond boundaries of subspecialties.

### Other therapy-oriented scales

In an attempt to make CDG more easily applicable in neurosurgery, Landriel *et al*.^8^ proposed a similar classification scheme for neurosurgical complications. Their complication grading system uses the same categories as CDG but modifies the labels. They found a lower percentage of complications and adverse events (14%), but a higher percentage of complications (CDG > 2) among these. Schiavolin *et al*.^9^ also used the Landriel classification and found a 6.1% rate of neurosurgical complications (CDG > 2) for a series of 327 patients undergoing surgery for spine degeneration or spinal tumor. When comparing these two studies with our data, the complications occurred at a similar rate.

### Other grading scales for complications in spine surgery

While reviewing the literature of complication scores in surgical patient populations we encountered a great variability of the usage of the term “complication”. Review of the literature shows that there is no clear definition of the terms “adverse event”, “complication” and “avoidable complication” and that sometimes these terms are used interchangeably.

For example Rampersaud *et al*.^10^ published a quantitative report and investigation on surgical adverse events in spine surgery and discussed the differences of adverse events and complications. They agreed that there is no clear-cut definition of either and in some cases differentiation between one or the other can be difficult. In their series they found surgical adverse events in 14% of 700 consecutive cases of which 77% did not require significant treatment or longer hospitalization. The other 23% of adverse events led to longer hospitalization or significant additional treatment and were classified as complications. Applying their complication score on our patients’ data we found almost the same relation between adverse events and complications. There were 24.6% of patients with adverse events (medical and surgical), which did not require significant treatment or longer hospitalization in 73.5% of cases (CDG score 1 and 2).

Another study by Houkin *et al*.^11^ reviewed neurosurgical procedures with regard to adverse events at two Japanese University Hospitals. They rated adverse events and complications retrospectively related to their cause and whether or not they were avoidable. They found adverse events in 28.3% of 643 consecutive neurosurgical interventions over a period of 2 years, which is comparable to the present study and the publication by Rampersaud *et al*. Among these 6 (3.3%) were considered avoidable and 2 (1.1%) caused by medical errors. Especially for spine surgery these numbers are not comparable as rating minor adverse events (like persisting postoperative pain or) into avoidable or not seems very difficult, especially in surgery for degenerative spine disease, in which improvement of quality of life is the goal of the surgical procedure. A postoperative myocardial infarction or postoperative persistent leg pain might both be an unavoidable adverse event or avoidable through patient selection (secondary medical diagnosis) and more extended surgical decompression.

Lebude *et al*.^12^ published a study to address the problem of the not well defined terms of “adverse event” and “complications” and conducted a survey on “defining complications” and sent a questionnaire out to 2000 spine surgeons of which 229 took part in the survey. They were presented patient cases with adverse events and were advised to rate them in the categories “minor complication”, “major complication” and “no complication”. Their results revealed differing surgeons’ opinions especially in cases, which showed “complications” that could as well be classified as adverse events or expected side effects of the surgical treatment.

While a number of complication grading schemes showed a correlation with clinical outcome in neurosurgical and spine surgical patient populations^8,10–12^, for a comparison of treatment between different centers, a transferable rating of complications and outcome would be desirable.

### Limitations

We limited the length of follow-up to 6 weeks as the focus of the study was on validation of CDG in regards to treatment costs during hospitalization, length of hospitalization and short term follow-up to document possible direct negative impact due to complications. For patients with micro-discectomies (78% of all procedures) this was the interval for postoperative routine visits for patients unless an unfavorable postoperative course warranted further visits. Furthermore, Patient-rated outcome measures (PROMS) were not used in this study because the primary goal of the study was to validate the CDG in a homogenous patient population to characterize the procedure in a surgical subspecialty.

## Conclusions

Our study has demonstrated the correlation between clinically relevant scales and the CDG grade of a complication, for the first time in a well-defined neurosurgical patient population. While the high correlation of CDG with cost seems self evident for a therapy-oriented grading system we could show correlation with treatment costs for the first time and it also correlated with clinical outcome. As a further advantage, CDG registers any deviation from the normal postoperative course and allows comparison between surgical specialties.
